# Impact of *APOE* gene polymorphisms on the lipid profile in an Algerian population

**DOI:** 10.1186/1476-511X-12-155

**Published:** 2013-10-25

**Authors:** Houssam Boulenouar, Sounnia Mediene Benchekor, Djabaria Naïma Meroufel, Sarah Aicha Lardjam Hetraf, Hadjira Ouhaibi Djellouli, Xavier Hermant, Benjamin Grenier-Boley, Imane Hamani Medjaoui, Nadhira Saidi Mehtar, Philippe Amouyel, Leila Houti, Aline Meirhaeghe, Louisa Goumidi

**Affiliations:** 1 Laboratoire de Génétique Moléculaire et Cellulaire, Université des Sciences et de Technologie d’Oran Mohamed Boudiaf, Oran, Algeria; 2 Département de Biotechnologie, Faculté des Sciences de la Nature et de la Vie, Université d’Oran, Oran, Algeria; 3 INSERM, U744; Institut Pasteur de Lille, Université Lille Nord de France, Lille, France; 4 Caisse Nationale des Assurances Sociales des travailleurs salariés, Clinique Spécialisée en Orthopédie et Rééducation des Victimes des Accidents de Travail, Oran, Algeria; 5 Faculté de Médecine, Université Djillali Liabes de Sidi Bel Abbes, Sidi Bel Abbes, Algeria; 6 LABoratoire des Systèmes d’Information en Santé, Université d’Oran, Oran, Algeria

**Keywords:** APOE, Polymorphism, Algerian population, Lipid parameters, Cardiovascular risk, General population sample, North Africa

## Abstract

**Background:**

The importance of apolipoprotein E (APOE) in lipid and lipoprotein metabolism is well established. However, the impact of *APOE* polymorphisms has never been investigated in an Algerian population. This study assessed, for the fist time, the relationships between three *APOE* polymorphisms (epsilon, rs439401, rs4420638) and plasma lipid concentrations in a general population sample from Algeria.

**Methods:**

The association analysis was performed in the ISOR study, a representative sample of the population living in Oran (787 subjects aged between 30 and 64). Polymorphisms were considered both individually and as haplotypes.

**Results:**

In the ISOR sample, *APOE* ϵ4 allele carriers had higher plasma triglyceride (*p*=0.0002), total cholesterol (*p*=0.009) and LDL-cholesterol (*p*=0.003) levels than ϵ3 allele carriers. No significant associations were detected for the rs4420638 and rs439401 SNPs. Linkage disequilibrium and haplotype analyses confirmed the respectively deleterious and protective impacts of the ϵ4 and ϵ2 alleles on LDL-cholesterol levels and showed that the G allele of the rs4420638 polymorphism may exert a protective effect on LDL-cholesterol levels in subjects bearing the *APOE* epsilon 4 allele.

**Conclusion:**

Our results showed that (i) the *APOE* epsilon polymorphism has the expected impact on the plasma lipid profile and (ii) the rs4420638 G allele may counterbalance the deleterious effect of the ϵ4 allele on LDL-cholesterol levels in an Algerian population.

## Background

Dyslipidemia (defined by elevated levels of fasting and post-prandial plasma triglyceride-rich lipoproteins, abnormally low high-density lipoprotein (HDL) levels and elevated low-density lipoprotein (LDL) concentrations) is associated with atherosclerosis and coronary heart disease (CHD) [1,2]. Coronary artery disease is a major cause of morbidity and mortality in both industrialized countries and developing countries, such as Algeria [3,4]. It has been estimated that there were 7.3 million deaths worldwide from ischemic heart disease in 2008 (12.4% of all mortality). Furthermore, it is predicted that CHD will still be the leading cause of death in 2020 [5,6].

Commonly studied intermediate traits associated with CHD include plasma levels of cholesterol (total cholesterol, LDL-cholesterol and HDL-cholesterol), body mass index (BMI) and blood pressure. These traits are influenced by a combination of genetic [7] and environmental factors (such as diet, alcohol and physical activity) [8-10].

The results of several meta-analyses have firmly established that the apolipoprotein E (*APOE*) epsilon polymorphism (defined by the rs7412 and rs429358 single nucleotide polymorphisms (SNPs)) is a genetic risk factor for CHD [11-14]. Indeed, APOE has an important role in the metabolism of lipoproteins and is a ligand for LDL-cholesterol and APOE receptors [15]. The epsilon polymorphism in the *APOE* gene leads to the generation of APOE2, APOE3 and APOE4 isoforms, which are coded by three codominant alleles (designated as ϵ2, ϵ3 and ϵ4). The three isoforms differ by an amino acid substitution at position 112 or position 158 in the 299-amino-acid peptide chain [16]. The isoforms interact differently with specific lipoprotein receptors and thus influence plasma cholesterol concentrations [17]. The *APOE* ϵ4 allele is associated with higher total and LDL-cholesterol levels and a higher risk of CHD, whereas the *APOE* ϵ2 allele is associated with the opposite (i.e. protective) effects in Caucasian populations [18-21].

Furthermore, two other *APOE* polymorphisms have been found to display associations with various metabolic traits. Firstly, the rs439401 SNP was associated with higher plasma triglyceride and lower plasma HDL-cholesterol concentrations in a meta-analysis of genome-wide association studies (GWAS) in 16 European cohorts [22]. Secondly, the rs4420638 SNP is reportedly associated with lower plasma HDL-cholesterol levels, higher total cholesterol and LDL-cholesterol levels and higher total cholesterol/HDL-cholesterol and LDL-cholesterol/HDL-cholesterol ratios [14,23-25].

Very few studies have investigated putative associations between the *APOE* epsilon polymorphism and plasma lipid levels in North African populations. Indeed, only two studies (in Moroccan and Tunisian populations) reported that the *APOE* ϵ4 allele is associated with higher plasma concentrations of total cholesterol and LDL-cholesterol, whereas the *APOE* ϵ2 allele shows the opposite association [26-28]. However, no data for the rs439401 and rs4420638 polymorphisms in these populations are available.

To the best of our knowledge, the relationship between *APOE* polymorphisms and plasma lipid and lipoprotein concentrations in an Algerian population has never previously been studied. We therefore decided to assess the relationships between *APOE* epsilon, rs439401 and rs4420638 polymorphisms and plasma lipid concentrations in a population sample from the city of Oran in north-west Algeria, the ISOR study.

## Results

### Genotype and allele distributions

The allele and genotype distributions of the *APOE* polymorphisms are presented in Table 1. There was no evidence of significant deviation from Hardy-Weinberg equilibrium in any distributions.

**Table 1:** **Genotype distributions of the** ***APOE*** **polymorphisms in the ISOR study**

**Polymorphism**		**n**	**(%)**
Epsilon	Genotype		
	ϵ2/ϵ2	3	(0.4)
	ϵ2/ϵ3	64	(8.7)
	ϵ2/ϵ4	3	(0.4)
	ϵ3/ϵ3	525	(71.8)
	ϵ3/ϵ4	124	(16.9)
	ϵ4/ϵ4	13	(1.8)
	Total	732	
	pH-W	0.07	
	Allele		
	ϵ2	73	(5.0)
	ϵ3	1238	(84.6)
	ϵ4	153	(10.4)
rs439401	Genotype		
	CC	288	(39.0)
	CT	335	(45.4)
	TT	115	(15.6)
	Total	738	
	pH-W	0.28	
	Allele		
	C	911	(61.7)
	T	565	(38.3)
rs4420638	Genotype		
	AA	596	(78.9)
	AG	149	(19.8)
	GG	10	(1.3)
	Total	755	
	pH-W	0.84	
	Allele		
	A	1341	(88.8)
	G	169	(11.2)

H-W: Hardy-Weinberg equilibrium.

### Linkage disequilibrium

**Figure 1:**
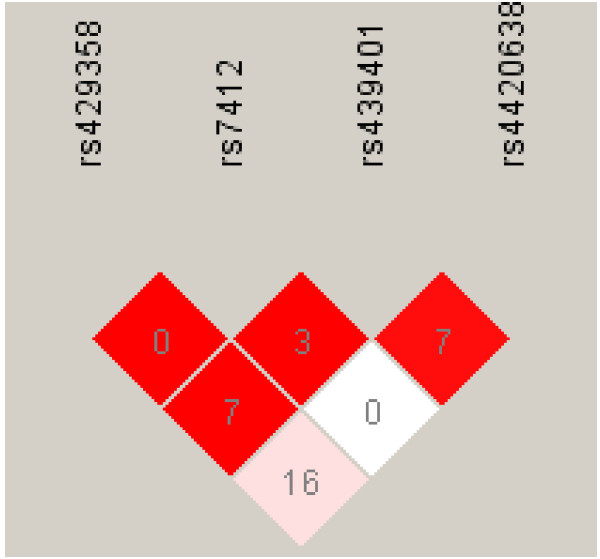
**Relative position in the *APOE *locus and LD values (D’ in black and white, r**^**2 **^**values) of the SNPs.** SNPs with the highest level of D’ are shown in black and those with the lowest level of D’ are shown in white. r^2^ values are indicated in the squares.

We evaluated the linkage disequilibrium (LD) between the *APOE* epsilon (rs429358 and rs7412), rs439401 and rs4420638 polymorphisms (Figure 1). The rs439401 and rs4420638 SNPs were not in LD with the *APOE* epsilon polymorphism (r^2^<0.16). So the analysis of all polymorphisms was investigated.

### Association studies

The various associations between the three *APOE* polymorphisms and the anthropometric phenotypes (weight, waist and hip circumferences and BMI), biochemical phenotypes (glucose, insulin, fasting plasma triglyceride, total cholesterol, HDL-cholesterol and LDL-cholesterol levels) and clinical phenotypes (SBP and DBP) were assessed (Tables 2–3).

**Table 2:** **Association between the** ***APOE*** **epsilon polymorphism and anthropometric, biochemical and clinical parameters in the ISOR study**

	**ϵ2ϵ2+ϵ2ϵ3**	**ϵ3ϵ3**	**ϵ3ϵ4+ϵ4ϵ4**	***p***^***a***^	***p***^***b***^
n	67	522	137		
Weight (kg)	74.6 ± 17.9	71.0 ± 14.2	70.7 ± 13.6	0.05	0.6
Waist (cm)	89.4 ± 13.3	87.6 ± 12.5	87.4 ± 11.6	0.33	0.81
Hip (cm)	103.0 ± 10.6	101.9 ± 9.7	101.0 ± 9.3	0.16	0.6
BMI (kg/m^2^)	27.3 ± 6.2	26.1 ± 5.1	25.5 ± 4.5	0.02	0.42
Waist-to-hip ratio	0.87 ± 0.08	0.86 ± 0.09	0.87 ± 0.08	0.93	0.71
Fasting glucose (mmol/L)*	4.98 ± 1.28	5.04 ± 1.30	5.20 ± 1.53	0.23	0.25
Fasting insulin (μIU/mL)*	9.18 ± 6.65	7.95 ± 5.48	8.65 ± 8.31	0.76	0.35
Triglycerides (mmol/L)^†^	1.34 ± 0.78	1.09 ± 0.44	1.24 ± 0.48	0.10	**0.0002**
Total cholesterol (mmol/L)^†^	4.25 ± 0.95	4.41 ± 0.86	4.63 ± 1.11	0.10	**0.009**
HDL-cholesterol (mmol/L)^†^	1.27 ± 0.34	1.26 ± 0.30	1.22 ± 0.32	0.40	0.06
LDL-cholesterol (mmol/L)^†^	2.47 ± 0.85	2.64 ± 0.82	2.88 ± 1.05	0.06	**0.003**
LDL-cholesterol/HDL-cholesterol ratio^†^	2.14 ± 1.07	2.25 ± 1.00	2.55 ± 1.23	0.18	**0.001**
SBP (mmHg)^††^	122.6 ± 16.3	120.6 ± 14.8	122.7 ± 17.8	0.95	0.13
DBP (mmHg)^††^	75.5 ± 8.8	76.6 ± 9.8	76.3 ± 11.1	0.83	0.38

Data are expressed as the mean ± SD. SBP: systolic blood pressure; DBP: diastolic blood pressure.

p values were adjusted for age, gender, smoking status and physical activity for anthropometric variables.

p values were adjusted for age, gender, BMI, smoking status and physical activity for biological and biochemical variables.

*p*^*a*^: p values obtained when comparing ϵ2ϵ2+ϵ2ϵ3 subjects with ϵ3ϵ3 subjects.

*p*^*b*^: p values obtained when comparing ϵ3ϵ4+ϵ4ϵ4 subjects with ϵ3ϵ3 subjects.

*subjects treated for diabetes were excluded (n=54).

^†^subjects treated with lipid-lowering drugs were excluded (n=36).

^††^subjects treated for hypertension were excluded (n=89).

**Table 3:** **Association between the** ***APOE*** **rs439401 and rs4420638 SNPs and anthropometric, biochemical and clinical parameters in the ISOR study**

	**rs439401**					**rs4420638**				
	**CC**	**CT**	**TT**	***p***^***1***^	***p***^***2***^	**AA**	**AG**	**GG**	***p***^***1***^	***p***^***2***^
n	288	335	115			596	149	10		
Weight (kg)	71.6 ± 14.5	71.5 ± 15.0	70.8 ± 13.6	0.87	0.68	71.7 ± 14.9	69.4 ± 13.4	67.7 ± 5.9	0.04	0.04
Waist (cm)	88.1 ± 12.6	87.7 ± 12.2	87.4 ± 12.4	0.83	0.66	88.0 ± 12.5	86.7 ± 11.9	83.5 ± 11.2	0.11	0.07
Hip (cm)	102.2 ± 10.0	102.0 ± 10.00	101.3 ± 9.1	0.56	0.45	102.2 ± 9.9	100.8 ± 9.3	97.0 ± 6.2	0.04	0.04
BMI (kg/m^2^)	26.0 ± 5.2	26.3 ± 5.1	26.0 ± 4.9	0.65	0.79	26.2 ± 5.2	25.4 ± 4.6	24.5 ± 2.9	0.05	0.08
Waist-to-hip ratio	0.86 ± 0.08	0.86 ± 0.10	0.86 ± 0.08	0.83	0.92	0.86 ± 0.08	0.86 ± 0.11	0.86 ± 0.11	0.99	0.64
Fasting glucose (mmol/L)*	5.04 ± 1.20	5.10 ± 1.52	5.06 ± 1.14	0.69	0.48	5.03 ± 1.26	5.20 ± 1.60	5.03 ± 0.70	0.18	0.34
Fasting insulin (μIU/mL)*	8.02 ± 6.09	8.57 ± 6.53	7.58 ± 5.62	0.72	0.7	8.07 ± 5.62	8.75 ± 8.28	6.61 ± 2.89	0.31	0.58
Triglycerides (mmol/L)^†^	1.20 ± 0.51	1.14 ± 0.50	1.07 ± 0.47	**0.005**	0.25	1.14 ± 0.49	1.19 ± 0.55	1.21 ± 0.41	0.06	0.89
Total cholesterol (mmol/L)^†^	4.50 ± 1.02	4.42 ± 0.86	4.35 ± 0.82	0.14	0.42	4.44 ± 0.90	4.43 ± 0.98	4.51 ±1.02	0.76	0.42
HDL-cholesterol (mmol/L)^†^	1.24 ± 0.31	1.26 ± 0.33	1.27 ± 0.27	0.29	0.41	1.25 ± 0.30	1.27 ± 0.34	1.17 ± 0.24	0.82	0.55
LDL-cholesterol (mmol/L)^†^	2.74 ± 0.97	2.66 ± 0.82	2.58 ± 0.82	0.11	0.31	2.69 ± 0.87	2.64 ± 0.88	2.78 ± 0.88	0.94	0.20
LDL-cholesterol/HDL-cholesterol ratio^†^	2.39 ± 1.15	2.38 ± 2.12	2.17 ± 0.88	0.31	0.19	2.37 ± 1.76	2.25 ± 1.02	2.36 ± 0.52	0.65	0.09
SBP (mmHg)^††^	121.4 ± 16.4	120.9 ± 14.8	121.1 ± 15.1	0.66	0.44	120.9 ± 14.8	121.0 ± 17.6	128.1 ± 18.9	0.36	0.51
DBP (mmHg)^††^	75.4 ± 10.1	75.9 ± 8.7	76.4 ± 8.6	0.14	0.09	75.7 ± 9.0	75.7 ± 10.1	78.1 ± 12.2	0.56	0.58

Data are expressed as the mean ± SD. SBP: systolic blood pressure, DBP: diastolic blood pressure.

*p*^*1*^: p values were adjusted for age, gender, smoking status and physical activity for anthropometric variables. p values were adjusted for age, gender, BMI, smoking status and physical activity for biological and biochemical variables.

*p*^*2*^: as above and additionally for *APOE* epsilon status.

*subjects treated for diabetes were excluded (n=54).

^†^subjects treated with lipid-lowering drugs were excluded (n=36).

^††^subjects treated for hypertension were excluded (n=89).

### The APOE epsilon polymorphism

No significant association could be detected when comparing ϵ2 allele carriers with ϵ3 allele carriers (Table 2). In contrast, ϵ4 allele carriers had significantly higher mean plasma triglyceride (*p*=0.0002), mean plasma total cholesterol (*p*=0.009), mean LDL-cholesterol (*p*=0.003) levels and LDL-cholesterol/HDL-cholesterol ratio (*p*=0.001) than ϵ3 allele carriers did (Table 2).

### The rs4420638 and rs439401 polymorphisms

No significant associations were detected for the rs4420638 SNP in the ISOR study (Table 3). In contrast, the T allele of rs439401 was significantly associated with lower plasma triglyceride levels (*p*=0.005) (Table 3). This association disappeared after further adjustment for the *APOE* epsilon polymorphism (*p*=0.25).

### Gene-environment analyses

As gender, menopausal status in women and lipid-lowering drugs use may significantly impact plasma lipid levels, we took into account these confounders and investigated associations between the *APOE* polymorphisms and plasma lipids, in men and women separately and in the non-menopausal women group. All previously described associations were replicated in each group, meaning that gender and menopausal status had no notable influence on the present associations (data not shown).

### Haplotype analysis

We explored the haplotype effects of the *APOE* epsilon (rs429358 and rs7412) and the rs439401 and rs4420638 SNPs on plasma LDL-cholesterol levels. First, we selected the most informative haplotype configuration. The best haplotype model included the *APOE* epsilon and the rs4420638 polymorphisms (*p*=0.002, with 4 d.f.). We then performed haplotype analysis (using Thesias [29]) for the *APOE* epsilon and rs4420638. Five haplotypes were inferred (ϵ3A, ϵ3G, ϵ4A, ϵ4G and ϵ2A) (Table 4). The test for an overall haplotype effect was significant (*p=*0.002).

**Table 4:** **Effect of the** ***APOE*** **haplotypes on plasma LDL-cholesterol levels in the ISOR study**

**Haplotypes**				
**rs429358/rs7412/rs4420638**	**ϵ/rs4420638**	**Frequency**	**Haplotype effect [95% CI] (mmol/L)**	***p***
TCA	ϵ3A	0.784	reference	-
TCG	ϵ3G	0.061	0.02 [-0.17 ─ 0.21]	0.74
CCA	ϵ4A	0.054	0.35 [0.18 ─ 0.51]	**0.0001**
CCG	ϵ4G	0.051	-0.05 [-0.27 ─ 0.17]	0.81
TTA	ϵ2A	0.050	-0.20 [-0.41 ─ -0.01]	**0.05**

Polymorphisms are ordered according to their position in the genomic sequence.

Values are the difference in means [95% CI] when compared with the ϵ3A reference haplotype (mean [95% CI]=1.33 [1.29 ─ 1.37] mmol/L) using Thesias software.

When compared with the most frequent (reference) ϵ3A haplotype, the ϵ4A haplotype was associated with higher LDL-cholesterol levels (Δ [95% CI]=+0.35 [0.18 ─ 0.51] mmol/L, *p*=0.0001), whereas the ϵ2A haplotype was associated with lower LDL-cholesterol levels (Δ [95% CI]=-0.20 [-0.41 ─ -0.01] mmol/L, *p*=0.05), confirming the respectively deleterious and protective impacts of the ϵ4 and ϵ2 alleles. The ϵ3G haplotype was associated with similar LDL-cholesterol levels (Δ [95% confidence interval (CI)]=0.02 [-0.17 ─ +0.21] mmol/L, *p*=0.74) compared with carriers of the ϵ3A haplotype; this result suggests that the rs4420638 G allele does not have an effect in an ϵ3 background. Interestingly and contrarily to what we observed for the ϵ4A haplotype, the ϵ4G haplotype was not associated with higher LDL-cholesterol levels, relative to the reference ϵ3A haplotype (Δ [95% CI]=-0.05 [-0.27 ─ -0.17] mmol/L, *p*=0.81). Hence, the G allele of the rs4420638 SNP may have counterbalanced the deleterious effect of the ϵ4 allele.

Similar haplotype results were obtained for plasma total cholesterol levels (data not shown).

## Discussion

The importance of APOE in lipid and lipoprotein metabolism is well established. However the impact of *APOE* polymorphisms has never been investigated in an Algerian population. So in this study, we assessed the relationship between three *APOE* polymorphisms (epsilon, rs439401 and rs4420638) and metabolic trait variations in a population from Oran, Algeria, the ISOR study. To the best of our knowledge, this was the first study to characterize associations of the three above-mentioned *APOE* gene polymorphisms with anthropometric, biochemical and clinical parameters in an Algerian population.

The frequency of the ϵ4 allele in Europe parallels the incidence of CHD and other diseases [11,30,31]. It follows a north-to-south gradient and ranges from more than 0.22 in Finland and Greenland [32-34] to less than 0.07 in Greece and Italy [35-37]. In our study, the ϵ4 allele frequency (0.10) was similar to values observed in other North African populations (around 0.10 in Moroccan populations [26,38] and around 0.08 in Tunisian populations [27,28]).

The minor allele frequency of the rs439401 SNP (located within the *APOE/APOC1* cluster) was 0.38 in the ISOR study. Similar frequencies have been reported in the literature and range from 0.33 to 0.36 [14,39].

For the rs4420638 SNP (situated lying 14 kb downstream of the *APOE* locus in the adjacent *APOC1* gene), the minor allele frequency (0.11) was markedly lower than the value observed in European samples [14,24,40].

In the ISOR study, the ϵ2 allele was not significantly associated with plasma lipid variations (0.06≤*p*≤0.18). This is probably due to too few of ϵ2 carriers (n=67). Only the ϵ4 allele showed an association with higher levels of total cholesterol and LDL-cholesterol. Furthermore, the ϵ4 allele was associated with higher concentrations of triglycerides, as previously observed by Kofler *et al.* in a study performed in the United Kingdom [41]. The APOE ϵ4 isoform (which binds preferentially to VLDLs) can interfere with plasma lipase activity or with the triglyceride removal system; this results in delayed lipolysis or clearance of plasma triglycerides in subjects bearing the ϵ4/ϵ3 phenotype [18]. Therefore, our results are consistent with previous studies [42-45]. In contrast with previous studies in multi-ethnic populations [41,46-48] but consistently with other studies in Tunisian subjects [27,28], we did not detect significant associations between the *APOE* epsilon polymorphism and HDL-cholesterol levels in the ISOR sample. Other studies conducted in Arab population samples are therefore needed to conclude.

Despite the deleterious effect of the ϵ4 allele on lipid parameters observed in the ISOR study, the lipid concentrations did not reach abnormal threshold values. A case–control study on the risk of myocardial infarction conducted in Oran in 2001 also showed that plasma concentrations of all lipid parameters were systematically lower in Algerian subjects than in Irish and French individuals [49].

We found significant association between the rs439401 SNP and triglyceride concentrations. This association has previously been observed in European GWAS of blood lipid levels [14,22]. However, this association disappeared after further adjustment for the *APOE* epsilon polymorphism, suggesting that the effect of rs439401 was overshadowed by the epsilon polymorphism.

In the ISOR study, the rs4420638 SNP was not significantly associated with any metabolic traits, contrary to what has been previously described [50]. These discrepancies could be explained by differences in the LD structure between rs4420638 and the *APOE* epsilon polymorphism in the Algerian and European populations (D’=0 and +16 with rs7412 and rs429358, respectively in the ISOR study versus D’=+66 and -69 with rs7412 and rs429358, respectively in the EUR panel of the 1000 Genomes database (December 2012 release, http://browser.1000genomes.org)). Therefore the effect of the rs4420638 SNP could be mainly due to the epsilon polymorphism in European samples but be independent in the ISOR study. This result should be investigated in other North-African populations.

Haplotype analysis was performed to better understand the information provided by the individual SNP analysis. This analysis showed that regarding plasma LDL-cholesterol (or total cholesterol) levels, the rs4420638 polymorphism could counterbalance the deleterious effect of the epsilon 4 allele.

## Conclusion

We showed that the impact of the *APOE* ϵ4 allele on the plasma lipid profile is similar in an Algerian population to what is known in other countries. It is noteworthy that the rs4420638 G allele could counterbalance the deleterious effect of the *APOE* ϵ4 allele on LDL-cholesterol levels in a North African population. Replications in larger studies are required.

## Methods

### Subjects

#### The ISOR (InSulino-résistance à ORan) study

The ISOR study was performed between 2007 and 2009. The study’s objectives and procedures were approved by the independent ethics committee at the Algerian National Agency for the Development of Health Research. The ISOR study was a population-based, cross-sectional study of a representative sample of 787 subjects (378 men and 409 women, aged between 30 and 64) recruited within the city of Oran. Subjects were selected at random from social security rolls. All subjects consented freely to participation in the study. A questionnaire on lifestyle (physical activity, tobacco use and alcohol intake), personal and family medical histories, current medication and socio-economic and educational levels was completed during a face-to-face interview. Anthropometric data were also recorded.

The level of physical activity was defined in quartiles as “none”, “low”, “medium” and “high” after summing exercise scores for sporting activities, walking, housework and physical activity at work.

In terms of tobacco use, participants were categorized as either smokers (i.e. individuals reporting at least one cigarette per day) or non-smokers. In the study questionnaire, subjects were asked to report their weekly consumption of wine, beer, and spirits. As there were very few drinkers (n=25, 3.2%), this variable was not taken into account in the final analysis.

The anthropometric measurements included height, body weight, waist circumference and hip circumference. Height and weight were measured while the subject was barefoot and lightly dressed. The BMI was calculated according to the Quetelet equation. Systolic and diastolic blood pressure values (SBP and DBP, respectively) were measured on the right arm with the subject in the sitting position, using a standard mercury sphygmomanometer. Measurements were made before and after completion of the questionnaire, with an interval of at least 10 minutes. The mean value of the blood pressure readings was considered in the final analysis.

After a 12 h overnight fast, two 15 ml blood samples were collected for each subject (in a disodium EDTA tube for subsequent DNA analysis and in a heparin tube for clinical chemistry tests).

A multichannel analyzer and dedicated kits (Humastar®, HUMAN Diagnostics, Wiesbaden, Germany) were used for the colorimetric, enzymatic measurement of cholesterol (kit: monotest cholesterol with cholesterol esterase, cholesterol oxidase and peroxidase), triglycerides (kit: peridochrom triglyceride with glycerol phosphate oxidase and peroxidase) and glucose (kit: glucose, glucose oxidase and peroxidase). Plasma LDL-cholesterol levels were calculated according to the Friedwald equation. High-density lipoprotein cholesterol levels were measured after sodium phosphotungstate/magnesium chloride precipitation of chylomicrons and VLDL and LDL-cholesterol and then centrifugation. Plasma insulin levels were measured in a microparticle enzyme immune assay running on an AxSYM analyzer (Abbott Laboratories, Abbott Park, Illinois, USA).

Genomic DNA was extracted from white blood cells by using the Stratagene® kit (Agilent Technologies, Les Ulis, France), according to the manufacturer’s protocol.

### Genotyping

The *APOE* SNPs (rs429358, rs7412, rs439401 and rs4420638) were genotyped using KASPar technology (KBioscience, Hoddesdon, UK) with the following probes: rs429358: [GACATGGAGGACGTG[C/T]GCGGCCGCCTGGTGC], rs7412: [GATGACCTGCAGAAG[C/T]GCCTGGCAGTGTACC], rs439401: [GCCGGCACTCTCTTC[C/T]CCTCCCACCCCCTCA], rs4420638: [TGCTACACTTTTCCT[A/G]GTGTGGTCTACCCGA]. The genotyping success rates ranged from 93% to 96%.

### Statistical analyses

Statistical analyses were performed with SAS 9.1 software (SAS Institute Inc., Cary, NC, USA). The Hardy-Weinberg equilibrium was tested using a χ^2^ test with one degree of freedom (d.f.).

Intergroup comparisons of means were performed with (i) a general linear model comparing ϵ2 carriers (ϵ2ϵ2 and ϵ2ϵ3 subjects) or ϵ4 carriers (ϵ4ϵ4 and ϵ4ϵ3 subjects) with ϵ3 carriers (ϵ3ϵ3 homozygotes) for the *APOE* epsilon polymorphism and (ii) an additive model for the rs439401 and rs4420638 polymorphisms. Subjects with the ϵ2ϵ4 genotype (n=3) were excluded from the analyses because of the possible opposing biological effects of the ϵ2 and ϵ4 alleles.

Data on triglycerides, glucose and insulin levels were log-transformed to obtain normal distributions. Estimated means were subsequently back–transformed for presentation in the tables.

For anthropometric variables, the confounding variables were age, gender, smoking status and physical activity. For biological and biochemical variables, the confounding variables were age, gender, BMI, smoking status and physical activity. For the rs439401 and rs4420638 polymorphisms, data were further adjusted for the *APOE* epsilon polymorphism coded in three genotypes as follows: ϵ2 carriers (ϵ2ϵ2 and ϵ2ϵ3 subjects); ϵ4 carriers (ϵ4ϵ4 and ϵ4ϵ3 subjects) and ϵ3 carriers (ϵ3ϵ3 subjects).

After Bonferroni correction, only associations with an uncorrected *p* value below 0.017 were considered to be statistically significant (i.e. 0.05 divided by the number of polymorphisms considered).

General linear models were used to investigate potential interactions by adding an interaction ((gender or BMI) x polymorphism) term.

Linkage disequilibrium figure and values were calculated with Haploview 4.2 (http://www.broadinstitute.org/scientific-community/science/programs/medical-and-population-genetics/haploview/haploview).

Haplotype frequencies derived from all studied polymorphisms were estimated independently of phenotype. A two-step haplotype analysis was performed. Firstly, in order to reduce the haplotype dimension and select the most informative, parsimonious haplotype configuration for the prediction of phenotypic variability, we applied the maximum likelihood model to all the possible 1 to *k*-loci combinations of polymorphisms that could be derived from the 4 *APOE* SNPs with the GridHaplo software [51]. Akaike’s information criterion (AIC) was calculated for each model (including a model with no polymorphisms) [52]. All AIC values were rescaled by subtracting the smallest AIC value obtained for the whole set of models. According to a rule derived by extensive Monte Carlo simulation, all models with a rescaled AIC ≤2 can be considered to be “equivalent” to the model with the lowest min *AIC*. The most parsimonious of the latter (corresponding to the minimal haplotype configuration) was selected. Secondly, haplotype analyses were performed using the Thesias software package (http://ecgene.net/genecanvas) [29]. Haplotype analyses were adjusted for age, gender, BMI, smoking status and physical activity level.

## Abbreviations

AIC: Akaike’s information criterion; APOE: Apolipoprotein E; BMI: Body mass index; CHD: Coronary heart disease; DBP: Diastolic blood pressure; DNA: Deoxyribonucleic acid; d.f.: Degree of freedom; GWAS: Genome-wide association studies; HDL: High-density lipoprotein; ISOR: InSulino-résistance à ORan; LD: Linkage disequilibrium; LDL: Low-density lipoprotein; SBP: Systolic blood pressure; SNP: Single nucleotide polymorphism; VLDL: Very low-density lipoprotein.

## Competing interests

The authors declare that they have no competing interests.

## Authors’ contributions

SMB, LH, NSM, PA, AM and LG designed research; SMB, LH, IMH, AM and LG conducted research; HOD, SLH, IMH, SMB and LH participated in the recruitment of subjects; LG built the database with the support of BGB; XH, DNM and HB performed the DNA extraction under the supervision of LG; HB and LG performed the statistical analyses; HB, SMB, AM and LG interpreted the results. IMH assayed biochemical parameters; HB wrote the paper under the supervision of SMB, AM and LG; HB, SMB, AM and LG had primary responsibility for final content. All authors read and approved the final manuscript.
